# An analytical approach to evaluate the performance of graphene and carbon nanotubes for NH_3_ gas sensor applications

**DOI:** 10.3762/bjnano.5.85

**Published:** 2014-05-28

**Authors:** Elnaz Akbari, Vijay Kumar Arora, Aria Enzevaee, Mohamad T Ahmadi, Mehdi Saeidmanesh, Mohsen Khaledian, Hediyeh Karimi, Rubiyah Yusof

**Affiliations:** 1 Centre for Artificial Intelligence and Robotics (CAIRO), Universiti Teknologi Malaysia, Kuala Lumpur, Malaysia; 2 Faculty of Electrical Engineering, Universiti Teknologi Malaysia, Johor Bahru, Malaysia; 3 Department of Electrical Engineering and Physics, Wilkes University, Wilkes-Barre, PA 18766, USA; 4 Faculty of Mechanical Engineering, Universiti Teknologi Malaysia, Johor Bahru, Malaysia; 5 Computational Nanoelectronic Research Group Faculty of Electrical Engineering, Universiti Teknologi Malaysia, Johor Bahru, Malaysia; 6 Malaysia–Japan International Institute of Technology (MJIIT), Universiti Teknologi Malaysia, Kuala Lumpur, Malaysia

**Keywords:** carbon nanotube (CNT), conductance, FET-based gas sensor, graphene

## Abstract

Carbon, in its variety of allotropes, especially graphene and carbon nanotubes (CNTs), holds great potential for applications in variety of sensors because of dangling π-bonds that can react with chemical elements. In spite of their excellent features, carbon nanotubes (CNTs) and graphene have not been fully exploited in the development of the nanoelectronic industry mainly because of poor understanding of the band structure of these allotropes. A mathematical model is proposed with a clear purpose to acquire an analytical understanding of the field-effect-transistor (FET) based gas detection mechanism. The conductance change in the CNT/graphene channel resulting from the chemical reaction between the gas and channel surface molecules is emphasized. NH_3_ has been used as the prototype gas to be detected by the nanosensor and the corresponding current–voltage (*I*–*V*) characteristics of the FET-based sensor are studied. A graphene-based gas sensor model is also developed. The results from graphene and CNT models are compared with the experimental data. A satisfactory agreement, within the uncertainties of the experiments, is obtained. Graphene-based gas sensor exhibits higher conductivity compared to that of CNT-based counterpart for similar ambient conditions.

## Introduction

There is a rapid growth in the development of sensors both in research and commercial applications. Our daily lives can be noticeably influenced by the development and advancement of miniature and/or portable gas sensors capable of accurately detecting analytes in real-time. Sensors with higher sensitivity and selectivity as well as faster response time are desired. Portability, remote operability and cost effectiveness are some of the features receiving considerable attention because of the ease of their implementation. Rapid advancement in nanoengineering as well as the production of faster and more compact integrated electronic components allow for these goals to be reached [1–6]. Nanotechnology is the study and application of materials with at least one dimension of the order of 1 to 100 nanometers, which is comparable to the de Broglie wavelength of carriers. Novel applications [7–9] are possible by exploiting the quantum waves in operation of these low-dimensional devices. New materials are being discovered in building novel sensors that can operate on the nanometer scale. Examples of these include graphene and carbon nanotubes (CNTs), as well as various semi-conductive nanowires and nanotubes [10–11]. Arora, Tan, and Gupta [12] have studied the carrier statistics of graphene and response of carriers to high electric fields. Arora and Bhattacharyya [13] have combined the carrier statistics of CNTs and discussed the band structure and its applications to quantum transport. In a recent paper [14], Chin et. al show how nanoelectronic parameters can be extracted from quantum conductance. In the next section, we advance these thoughts as we design the sensor made out of graphene and CNT.

### Carbon nanotubes and graphene

CNTs were first discovered by Sumio Iijima in 1991 [15] and have been extensively studied ever since. A single-walled carbon nanotube (SWCNT) is formed by rolling up a honeycomb lattice of a single atomic carbon sheet, i.e., graphene along a specific axis [16], known as chiral direction. The diameter of a typical CNT is around a few nanometers and its length can be over a micrometer, making it distinctly one-dimensional (1D) in its conductance with propagating quantum waves in the quasi free direction along the length of tube. Standing quantum waves are formed in the periphery of the tube forming the cylinder. A CNT is known to have a very high electrical and thermal conductivity as well as a high Young's modulus giving it the mechanical strength. The applications of CNTs are broad due to their compact structure and include transistors, sensors, solar cells, fuel cells, etc. [17].

**Figure 1:**
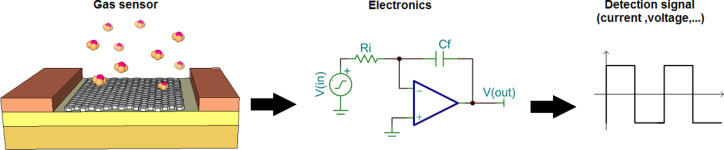
Schematic of a gas sensor.

Andre Geim and Konstantin Novoselov [18] discuss several applications of graphene, as one of the allotropes of carbon, which can be described as a single atomic layer of graphite. In this material, a two-dimensional honeycomb structure of sp^2^-bonded carbon atoms is tightly packed in a lattice structure [18]. Due to its zero bandgap energy, graphene has a high electron mobility at room temperature. The electron transfer in graphene is 100 times faster than that in silicon. A zero band gap with massless Dirac fermions makes graphene theoretically lossless, making it a perfect two-dimensional (2D) semiconductor [19–21]. Due to the abovementioned outstanding characteristics, graphene and CNT are being used as possible candidates for high performance gas sensors. When integrated in the sensor circuit and exposed to an analyte gas as illustrated in Figure 1, the detection signals are obtained through the changes in the *I*–*V* characteristics of graphene/CNT. Operational amplifiers amplify these signals that can be converted to digital format for digital signal processing.

### FET-based structure

**Figure 2:**
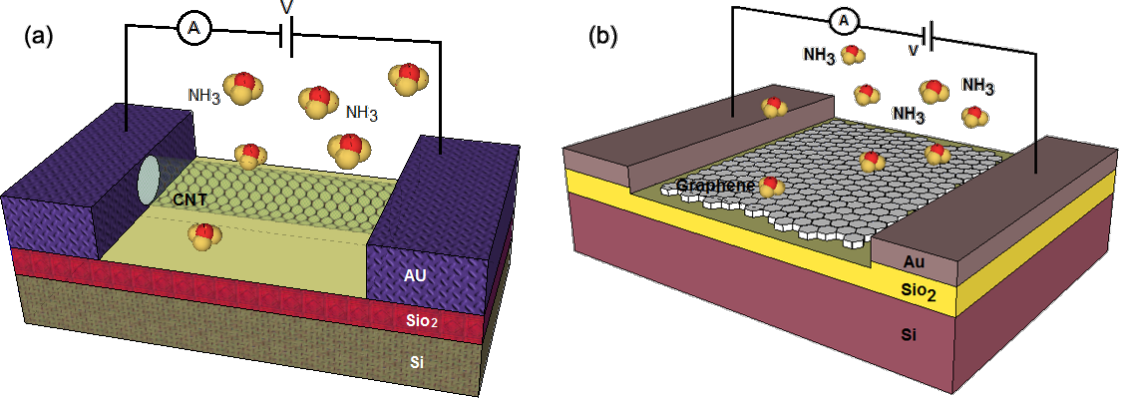
FET-based structure for gas sensor with (a) CNT channel and (b) graphene channel.

As presented in Figure 2, the structure of the proposed gas sensors that use CNT/graphene as the conducting channel looks quite similar to the conventional metal-oxide semiconductor field effect transistor (MOSFET), which comprises source and drain electrodes with the gate insulator inducing the channel of carriers and a silicon back gate [22–23] to augment the carrier density or adjust the threshold voltage. A CNT/graphene channel connects the source and the drain electrodes, and the gate is separated from the channel by a dielectric barrier layer. In most studies, silicon is used as the back gate while SiO_2_ is employed to act as a dielectric layer [23–24]. When gas molecules are in contact with the surface of CNT/graphene, the carrier concentration will change due to the variability of the current in the drain and the source, which is a measurable parameter [5,25–29]. The best gas sensor has a high sensitivity and is capable of sensing even one atom or molecule of gas [3,30]. Numerous recent theoretical studies on gas molecular adsorption on CNT/graphene have been reported for NO_2_, H_2_O, NH_3_, CO, and NO molecules that are physically adsorbed on pristine CNT/graphene [31–32].

**Figure 3:**
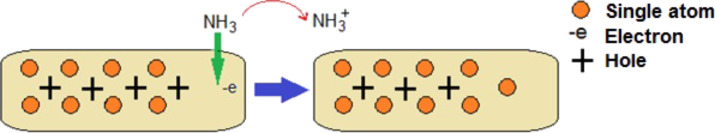
Schematic of the NH_3_ sensing mechanism based on the gas adsorption phenomenon.

Figure 3 illustrates a schematic representation of CNTs when electron-donating NH_3_ gas molecules are in the atmosphere around the sensor. Under such conditions, NH_3_ molecules are adsorbed on the surface of the CNT channel and donate electrons to it. This process causes a quite significant change in the electrical properties of the CNT. These strong adsorption effects stem from the inherent properties of gas molecules and the bonding characteristics between these molecules and the CNT [33–34]. It is always important to obtain p-type and/or n-type semiconducting CNT to incorporate them in a complementary logic. A p–n junction is a result of this complementarity. n-Type and p-type nanoscale field effect transistors can be formed for implementation by applying positive or negative gate voltage and can be useful from the application perspective [35].

Gas molecules can modulate the electronic structure of graphene in diverse ways. The adsorption of CO_2_ and O_2_ converts the system to p-type semiconductor while the adsorption of NH_3_ leads to n-type behavior. Similar to CNTs, these rich adsorption effects are caused by the intrinsic property of the gas molecules and the bonding characteristics between gas molecules and graphene [36]. The resulting p-type and n-type semiconducting behavior might be detected in experiment by applying and modulating gate voltage. Among all gas molecules considered, obviously NH_3_ molecule adsorption can greatly enhance the conductance [32,36].

## Proposed model for CNTs

We attempt to model the CNT conductance by considering the energy dispersion relation, and deriving the final model by using the Taylor series expansion near the Fermi points, as follows [37–38]:

[1]
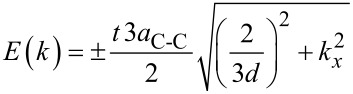


where the (±) sign has been included to account for the valence and conductance bands. *a*_C-C_ = 0.142 nm represents carbon–carbon bond length, *d* denotes CNT diameter and *t* = 2.7 eV is the nearest neighbour C–C tight binding overlap energy. For the first band gap energy we can simply write *E*_G_ = (2*a*_C-C_·*t*/*d*) = 0.8 eV·nm/*d* (nm). In addition, since the band structure is parabolic near the *k* = 0 points, we can write for the energy:

[2]
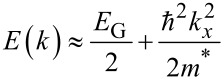


where 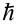 is the reduced Planck’s constant, *k_x_* represents the longitudinal wave vector component along the length of the tube and m* denotes the effective mass of the CNT effective mass depending on the tube diameter [39–40]. The number of conduction channels can be written as:

[3]
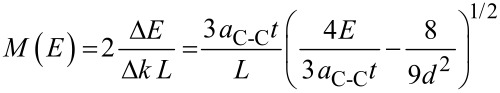


where *L* denotes the channel length. Two major factors contribute to the conductance effect on large channels, enabling it to follow the Ohmic scaling law based on the Landauer formula. The first factor, which is independent of length, is the interface resistance. The second one results from the fact that the relation between the conductance and the width is nonlinear and is dependent upon the number of modes in the conductor. However, these modes are the quantized parameters in the Landauer formula in which both factors are interrelated as demonstrated below [41]:

[4]
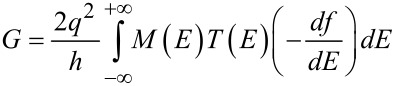


where *h* is the Planck’s constant, *q* denotes the electron charge and *T* is the transmission probability of an electron injected through the channel approximated as *T*(*E*) = 1 in ballistic channels [42]. *f*(*E*) is the Fermi–Dirac distribution with *df*/*dE* exploding to a delta function near the Fermi energy for degenerate statistics. The conductance can be obtained as follows [43]:

[5]
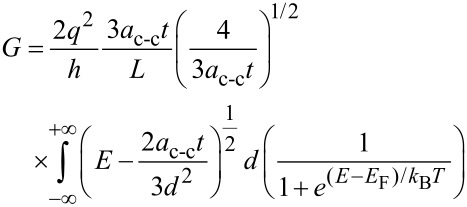


Equation 5 can be re-written as:

[6]
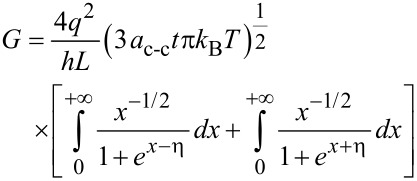


where *x* = (*E* – *E*_G_)/*k*_B_*T* and the normalized Fermi energy is given by η = (*E*_F_ – *E*_G_)/*k*_B_*T*. This equation can be numerically solved by applying the partial integration method [44–46]. The general model for the conductance of carbon nanotube-based gas sensor can be derived similar to that of silicon-based model proposed by Gunlycke [47].

[7]
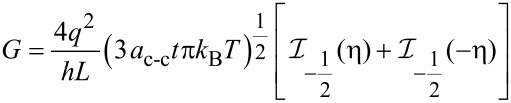



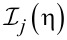
 is the Fermi–Dirac integral of the order *j*. The conductance characteristic demonstrates the performance of NH_3_ gas sensor based on a CNT nanostructure. It has been revealed that when the CNT gas sensor is exposed to NH_3_, the conductance changes [48]. We have proposed a model based on the reported experimental data and the relationship between conductance, gas concentration and temperatures as follows [49]:

[8]
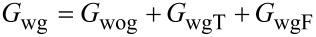


When the sensor is exposed to the gases in different temperatures, we can define three components for conductance, namely *G*_wog_, *G*_wg_**_T_** and *G*_wgF_. The first component *G*_wog_, is the conductance without the presence of gas. *G_wg_****_T_*** is defined as the conductivity changes in the presence of gas depending on the temperature and the last component, *G*_wgF_, is based on different values of gas concentration at a constant temperature [49]. The conductance changes with temperature and various concentrations when CNT gas sensor is exposed to NH_3_. *E*_G_ is dependent on temperature and gas concentration. Consequently, we can write:

[9]
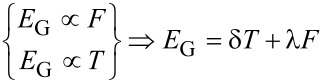


Writing η = (*E*_F_ – *E*_G_)/*k*_B_*T* explicitly, we obtain

[10]
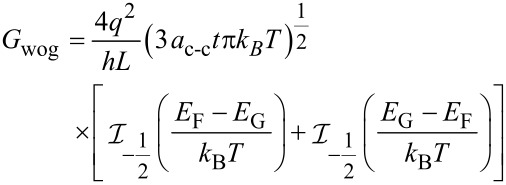


Equation 9 and Equation 10 are combined to obtain the conductance of gas sensor as:

[11]
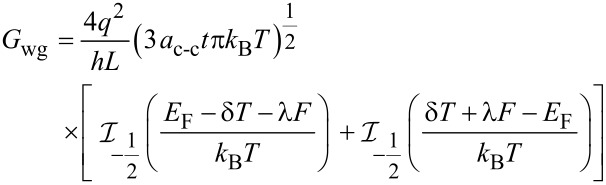


The Fermi–Dirac integral plays a significant role in the modeling of the behavior of the semiconductor. So, the following expansion of the Fermi–Dirac integral is taken into consideration:

[12]
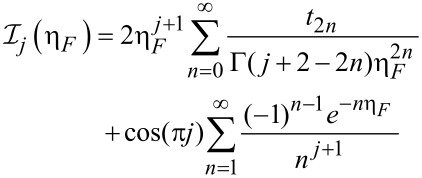


where *t*_0_ = 1/2, 
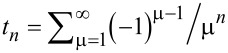
 = (1 – 2^1−^*^n^*)ζ(*n*), and ζ(*n*) is the Riemann Zeta function. In the degenerate limit (η >> 0), which is the operation regime for the nanometer-scale devices, the expressions for the Fermi–Dirac integral can be obtained from Equation 12 as 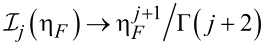. Accordingly, the Fermi–Dirac integral of order –1/2 can be simplified as [50]:

[13]
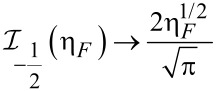


Based on the current–voltage characteristic of graphene-based FET devices, the gas sensor performance can be evaluated through Equation 14. Assuming that the source and substrate terminals are kept in ground potential, by applying a small voltage between source and drain (*V*_DS_), the channel region experiences a flow of electrons. Moreover, the relationship between current and conductance can be replaced by Fermi–Dirac integral of the general conductance model of SWCNT as:

[14]
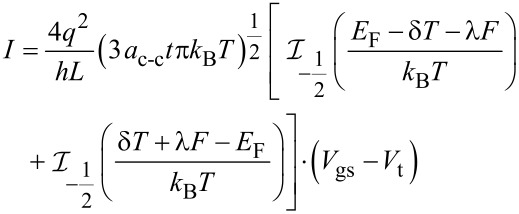


where *V*_gs_ is the gate–source voltage and *V*_t_ is the threshold voltage.

## Proposed model for graphene

The underlying operational principle in MOSFET is based on the electron flow between the source and drain electrodes, which can be controlled by the gate voltage. According to Landauer formula, there is a direct proportionality between conductance *G* and the transmission probability *T* of carriers from one electrode to another demonstrated by [41]:

[15]
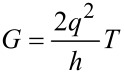


Taylor expansion is used to investigate a parabolic relationship involving energy and wave vector [51]:

[16]
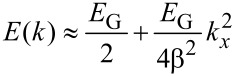


where β is the quantized wave vector given in [52]. The wave vector in the parabolic part of the band energy can be extracted as:

[17]
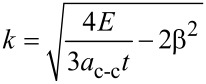


The conductance on large channel following the Ohmic scaling law based on Landauer formula can then be obtained as:

[18]
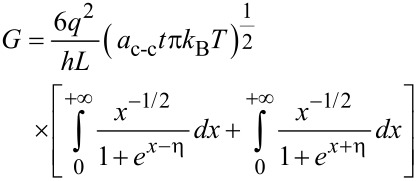


where x = (*E* – *E*_G_)/*k*_B_*T* and the normalized Fermi energy is η = (*E*_F_ – *E*_G_)/*k*_B_*T*. The performance of NH_3_ gas sensor based on graphene nanostructure is demonstrated by its conductance characteristic. It has been shown that the conductance changes when the graphene gas sensor is exposed to NH_3_ [53].

The corresponding formula equating the *I*–*V* characteristic of the graphene channel can then be written as:

[19]
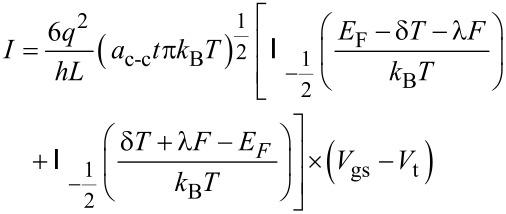


## Results and Discussion

**Figure 4:**
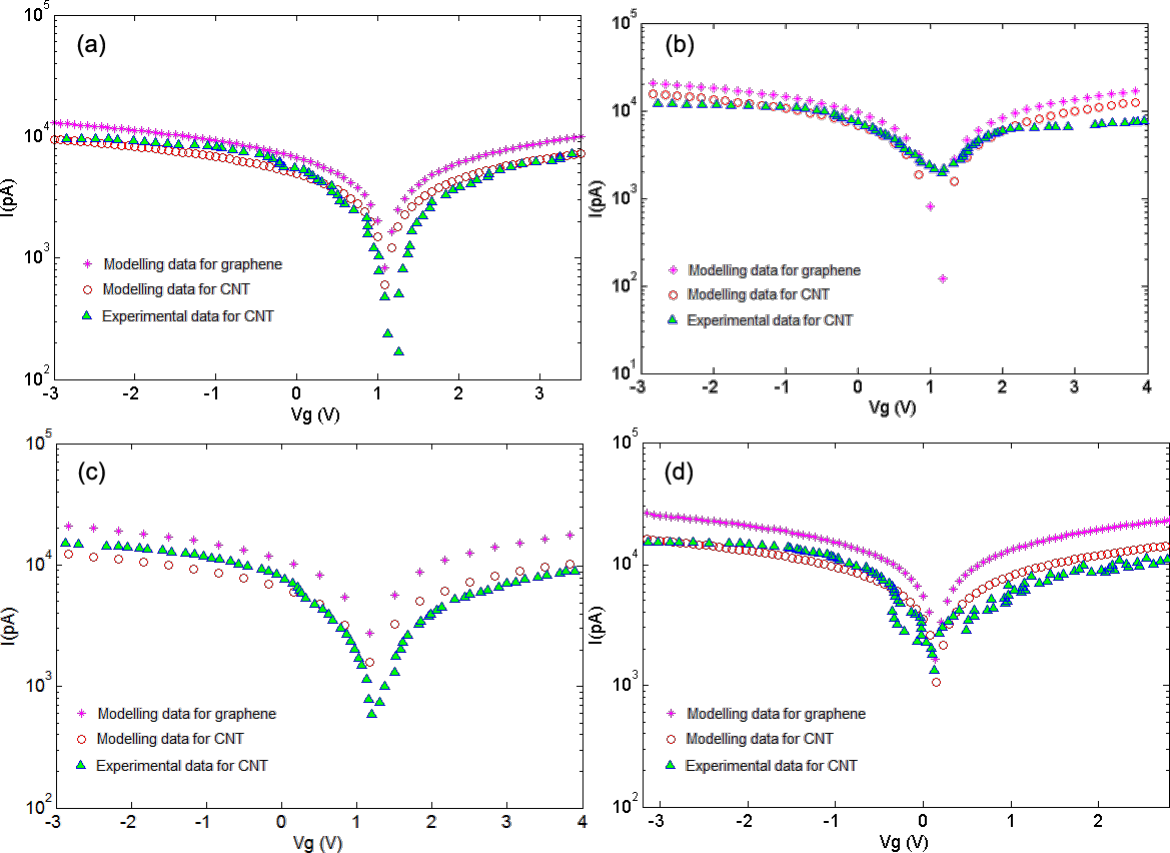
*I*–*V* characteristics of graphene and CNT after exposure to NH_3_ under *F* = 500 ppm at (a) *T* = 25 °C, (b) *T* = 50 °C, (c) *T* = 100 °C, (d) *T* = 150 °C.

Figure 4 illustrates the assessments of the gas sensor performance based on CNT and graphene nano-structures by considering their current–voltage characteristics when they are exposed to NH_3_ [53]. Also shown is the experimental data [53]. The agreement is good except near the minimum, for which the Dirac point is shifted to positive gate voltage.

Charge transfer is involved within the sensing mechanism of graphene and CNT-based gas sensors. This occurs during the interaction of gas molecules with the graphene and CNT surfaces. The conductivities of both channel media are modified through this interaction. The phenomenon is likely to occur as a result of the interaction of NH_3_ molecules with the carbon on the surface of graphene/CNT. Thus, electrons move from NH_3_ molecules to these materials. Figure 4 illustrates the *I*–*V* characteristics of the graphene/CNT gas sensors corresponding to temperatures of 25, 50, 100, and 150 °C, respectively. At the first three temperatures, for the minimum values of current, there have not been significant changes in the gate voltage. This can imply that at temperatures below 150 °C, the gas is reluctant to be adsorbed on graphene/CNT. However, the currents corresponding to each gate voltage value have risen at higher temperatures in all these four cases. Also, as shown in Figure 4d, at 150 °C the gate voltage becomes more negative. NH_3_ is an electron donating agent and it leaves electrons on the channel. This causes the graphene/CNT Fermi level to move toward their conduction band edges, making the threshold voltage *V*_th_ more negative. Thus, it can be said that this shift toward negative gate voltage is caused by the adsorption of NH_3_ on the graphene/CNT channel at this temperature.

**Figure 5:**
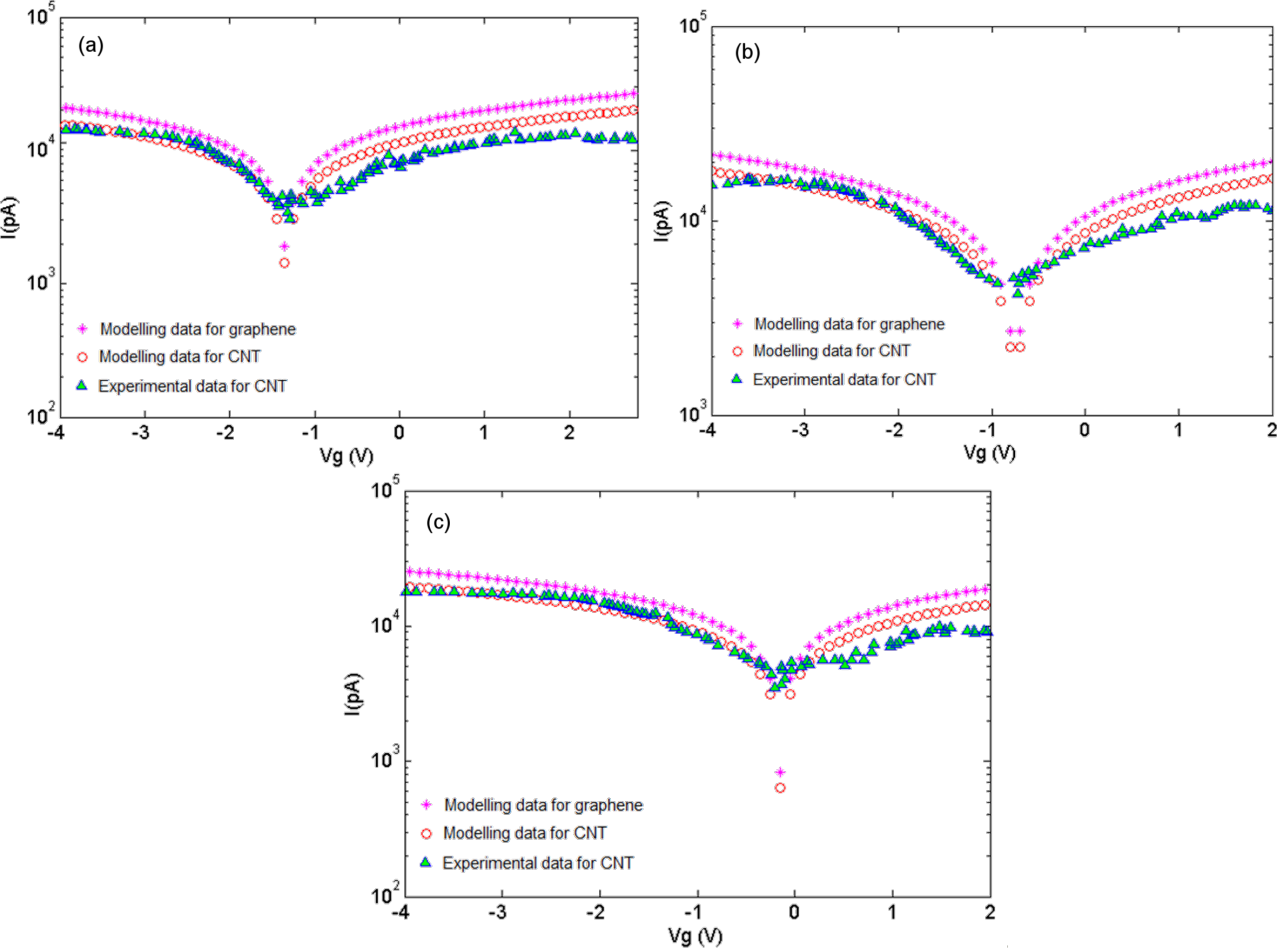
*I*–*V* characteristics after exposure to NH_3_ for graphene and CNT at *T* = 200 °C and under (a) *F* = 100 ppm, (b) *F* = 200 ppm, (c) *F* = 500 ppm.

Figure 5 illustrates the *I*–*V* characteristics of the proposed models for graphene and CNT in comparison with results for a CNT based experiment. An increase in the current can be associated with the charge transfer between NH_3_ molecules and graphene/CNT where the NH_3_ molecules operate as the donor. This phenomenon is also known as chemical doping by gas molecules. The sensitivity can be observed from the response of graphene/CNT-based gas sensors under 100 ppm, 200 ppm and 500 ppm NH_3_. A decreasing trend in the gate voltage similar to that for 150 °C can be seen at 200 °C. It can be concluded that at temperatures above 150 °C, the NH_3_ adsorption and the consequent electron donating behavior increases, which causes a further shift of the gate voltage toward negative values. The figure gives a clear illustration of the fact that there is a good agreement between the proposed models and extracted data [53]. In the suggested models, different values of temperature and gas concentration are demonstrated in the terms of the parameters δ and λ, respectively, as presented in Table 1.

**Table 1:** Different temperature and concentration values with respective parameters δ and λ.

*T* (°C)	*F* (ppm)	δ	λ

25	500	−3.65	0.027
50	500	−2.35	0.027
100	500	−1.45	0.027
150	500	−0.95	0.027
200	100	−0.7	0.005
200	200	−0.7	0.012
200	500	−0.7	0.027

Referring to the analytical models, δ has been introduced as the temperature control parameter obtained by iteration. The analytical models in our study show that the rate of changes in conductivity depending on temperature can be expressed by the following equation:

[20]
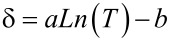


Here, parameters a and b are calculated to be *a* = 0.0138 and b = 0.0595. The parameter λ has been defined as a control parameter of gas concentration also calculated by iteration and shows that the rate of changes in conductivity depends on gas concentration, for which the equation can be written as:

[21]
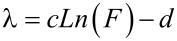


where the constants are calculated in the same manner to be *c* = 1.4129 and *d* = 8.0494.

## Conclusion

Outstanding properties such as high sensitivity as well as remarkable carrier transport features make both graphene and CNTs promising candidates for use in nanosensors. It has been observed that these materials experience a measureable change in conductance levels when exposed to NH_3_. This interesting feature has been suggested to be employed in gas detection systems. Two control parameters, i.e., the temperature control parameter (δ) and gas concentration control parameter (λ) have been introduced. A comparative analysis between the FET-based models for graphene/CNT sensor structures has been carried out, in which the latter has been validated by an experimental work by Peng et al. [53]. Aiming to minimize the error, the coefficients δ and λ are calculated by iteration. The *I*–*V* characteristics of the gas sensors are considered for the comparative study under exposure to different NH_3_ concentrations and temperatures. Finally, the comparison between the *I*–*V* characteristics of graphene and CNTs under similar conditions shows that graphene exhibits a higher conductivity than CNTs.
